# The potential of the gut microbiome for identifying Alzheimer’s disease diagnostic biomarkers and future therapies

**DOI:** 10.3389/fnins.2023.1130730

**Published:** 2023-04-27

**Authors:** Yu Zhan, Murad Al-Nusaif, Cong Ding, Li Zhao, Chunbo Dong

**Affiliations:** ^1^Department of Neurology, First Affiliated Hospital, Dalian Medical University, Dalian, China; ^2^Liaoning Provincial Key Laboratories for Research on the Pathogenic Mechanism of Neurological Disease, First Affiliated Hospital, Dalian Medical University, Dalian, China; ^3^The Center for Gerontology and Geriatrics, Dalian Friendship Hospital, Dalian, China

**Keywords:** Alzheimer’s disease, microbiome-gut-brain axis, biomarkers, traditional Chinese medicine, gut microbiome

## Abstract

Being isolated from the peripheral system by the blood–brain barrier, the brain has long been considered a completely impervious tissue. However, recent findings show that the gut microbiome (GM) influences gastrointestinal and brain disorders such as Alzheimer’s disease (AD). Despite several hypotheses, such as neuroinflammation, tau hyperphosphorylation, amyloid plaques, neurofibrillary tangles, and oxidative stress, being proposed to explain the origin and progression of AD, the pathogenesis remains incompletely understood. Epigenetic, molecular, and pathological studies suggest that GM influences AD development and have endeavored to find predictive, sensitive, non-invasive, and accurate biomarkers for early disease diagnosis and monitoring of progression. Given the growing interest in the involvement of GM in AD, current research endeavors to identify prospective gut biomarkers for both preclinical and clinical diagnoses, as well as targeted therapy techniques. Here, we discuss the most recent findings on gut changes in AD, microbiome-based biomarkers, prospective clinical diagnostic uses, and targeted therapy approaches. Furthermore, we addressed herbal components, which could provide a new venue for AD diagnostic and therapy research.

## 1. Introduction

Alzheimer’s disease (AD) is the most common neurodegenerative disease of cognitive decline in people over 65 years old, accounting for 60–70% of cases (Daniilidou et al., 2011; 2022 Alzheimer’s disease facts and figures, 2022; Dementia, 2022). AD is characterized by amyloid-β protein (Aβ) deposition, abnormal phosphorylation aggregation of the microtubule-associated protein tau, neuroinflammation, oxidative stress, and synaptic dysfunction, which are reflected by microglial reaction and increased cytokine production (Braak and Braak, 1991; Heneka et al., 2015; Lepeta et al., 2016; Butterfield and Halliwell, 2019; Bairamian et al., 2022; Paolicelli et al., 2022). The rapidly expanding effect of AD on socioeconomics and health care is becoming a global health issue. The global prevalence of dementia is expected to reach 78 million people by 2030 and is projected to reach 152 million by 2050 (ADI-Dementia Statistics, 2022). The gut microbiome (GM) comprises various microorganisms, including several species of viruses, bacteria, protozoa, and fungi (Cryan et al., 2019). GM displays vast diversity among different population groups and significantly influences human health, disease state, and overall well-being (Zmora et al., 2019). The microbiome-gut-brain axis (MGBA) links the GM and the brain via epigenetic, neural, and humoral pathways (Roy Sarkar and Banerjee, 2019; Liu et al., 2020; Nagu et al., 2021). Dysbiosis occurs when the GM is abnormally altered (Liu et al., 2020), leading to neurological disorders such as AD (Spielman et al., 2018; Sherwin et al., 2019; Zhu et al., 2021; Chidambaram et al., 2022).

This devastating and progressive disease’s neuropathological evaluation has evolved in preclinical and clinical fields (Zhang P.-F. et al., 2022). The diagnostic criteria for AD were refined in 2011 and 2018 while affirming the use of biomarkers and the new ability to describe the preclinical phase of the disease (Jack et al., 2018). Specific intestinal changes in pre-AD have been suggested to be involved in pre-AD pathology (Guo et al., 2021; Sheng et al., 2021). Therefore, gut-associated biomarkers may be a promising alternative or complementary tool for assessing disease conditions (O’Toole and Jeffery, 2015). Indeed, gut microbial-derived biomarkers have reported strong predictive and differential diagnostic power when used in studies such as psychological and neurodegenerative disorders (Qian et al., 2020; Lucidi et al., 2021). Furthermore, growing evidence demonstrates that MGBA is involved in the pathogenesis of AD. Hence, it might be modulated to regulate metabolite levels and remodel the gut barrier balance, improving cognition and providing a novel therapeutic approach. Consequently, it is imperative to explore in depth the critical role of the GM in the pathogenesis of AD and to evaluate further the value of gut biomarkers in early diagnosis, progression monitoring, and potential target therapy of the disease. Here, we review (1) gut involvement in peripheral and central nervous system (CNS) crosstalk in AD, (2) characteristic changes in the GM of AD and microbiome-based biomarkers, and (3) microbiome-potential targeted therapies to provide new ideas for early diagnosis of AD and directions for therapeutic drug development.

## 2. Crosstalk between the peripheral and central systems and alterations in AD

Epigenetics associated with AD include histone modifications and deoxyribonucleic acid (DNA) methylation, regulated by acetylases and methylases (Esposito and Sherr, 2019). Metabolites produced by the microbiome can inhibit histone deacetylases and other epigenetic marks (Maslowski and Mackay, 2011) and regulate inflammatory responses in the CNS (Bolduc et al., 2017). Moreover, they impact the acetylation and methylation of DNA and histones, allowing GM to trigger the expression of MHC class II molecules (Reid et al., 2017; Ye et al., 2017). Another aspect is microglia, myeloid innate immune cells in the CNS (Salter and Stevens, 2017). Recent preclinical, genetic, and bioinformatics data suggest that its reaction is accompanied by pathological changes in AD and is regulated by the microbiome (Colombo et al., 2021; Zhou et al., 2022). Downregulation of homeostatic genes *in vivo* and overexpression of recognized AD-associated genes, including apolipoprotein E and tyrosine protein tyrosine kinase binding protein, are linked to the transition to disease-associated microglia (Keren-Shaul et al., 2017; Leng and Edison, 2021). The identification of a correlation between AD and mutations in trigger receptor genes expressed on myeloid 2 (TREM2) and the myeloid cell surface antigen sialic acid binding Ig-like lectin 3 molecule supports for the first time the link between immune changes and AD pathogenesis (Bradshaw et al., 2013; Guerreiro et al., 2013; Jonsson et al., 2013). Functional TREM2 expression is downregulated in some cases of late-onset AD and may exacerbate intestinal microbial metabolites’ endotoxin-induced pro-inflammatory responses (Zhong et al., 2015), causing defective antibody clearance (Xiang et al., 2016).

### 2.1. The relationship between the gut microbiome and neuroinflammation and synaptic plasticity in AD

Gut microbiome may lead to increased neuroinflammation and decreased synaptic plasticity, both of which are thought to contribute to the development and progression of AD (Jiang et al., 2021). Gut microbiomes can alter the composition of the immune cells in the brain, leading to an increase in neuroinflammation (Erny et al., 2015). For example, studies in animal models have shown that colonization with certain types of gut bacteria can lead to microglial activation. Deficiency of the gut microbiome first affects the microglial cell transcriptome, which primarily regulates the interconversion of microglial cell subpopulations, and transcriptome changes are primarily associated with AD (Huang et al., 2023). Studies have shown that changes in the gut microbiome can alter synaptic plasticity in the brain. For example, as recently reviewed, germ-free mice (raised without exposure to microorganisms) have impaired synaptic plasticity compared to conventionally-raised mice (Glinert et al., 2022).

Synaptic dysfunction and microglia may interact through the microbiome (Bairamian et al., 2022), and synaptic dysfunction may precede symptoms of cognitive impairment (Selkoe and Hardy, 2016), as evidenced by diminished long-term potentiation (LTP) and long-term depression, which are closely related to synaptic plasticity (Bairamian et al., 2022). In addition, animal model studies have identified gut microbiome metabolites that regulate LTP in physiological and AD-related pathologies mechanisms, including influencing the expression of pre-and postsynaptic neuronal membrane receptors and membrane genes that further influence ion channels and thus affect synaptic activity (Albuquerque et al., 2015; Tong et al., 2015). These findings suggest a complex relationship between the gut microbiome, neuroinflammation, synaptic plasticity, and AD development. While much more research is needed to fully understand these relationships, the emerging evidence suggests that targeting the gut microbiome may represent a promising avenue for developing new treatments for this devastating disease.

### 2.2. Crosstalk pathways and molecules

**Figure 1:**
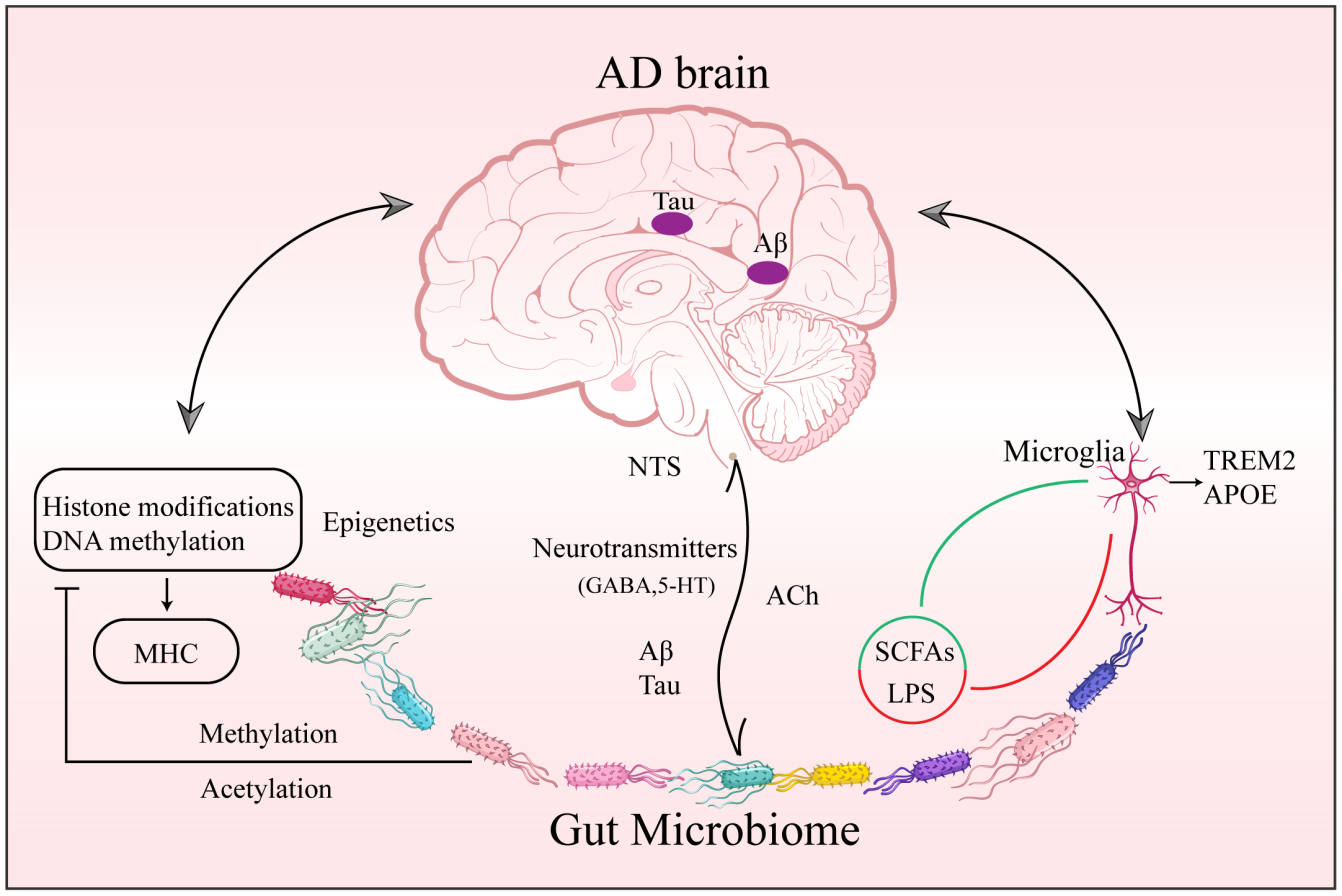
Gut Microbiome epigenetic, peripheral, and central pathology of AD. Changes in gut microbial composition, abundance, and metabolite diversity are involved in AD through epigenetic regulation of CNS function. (1) through acetylases, methylases, and deacetylases, affecting acetylation and methylation of DNA and histones; and (2) through microglia upregulation of APOE and downregulation of TREM2 associated with AD pathogenesis. Abbreviations: AD: Alzheimer’s disease; CNS: central nervous system; DNA: deoxyribonucleic acid; APOE: apolipoprotein E; TREM2: trigger receptor genes expressed on myeloid 2.

Inflammation in AD is not confined to neuroinflammation but includes the complex signals between microbial involvement in peripheral and central interaction. This crosstalk may comprise many neuronal, endocrine, immunological, and metabolic pathways. To be more precise, by exploiting the MGBA. This section concentrates on interacting with the brain through neurological and humoral pathways (Abdel-Haq et al., 2019). Figure 1 concisely summarizes the role that the gut microbiome plays in AD in terms of epigenetics as well as central and peripheral crosstalk.

#### 2.2.1. Vagus nerve signaling

The vagus nerve is a crucial neuronal pathway within the MGBA. Its functionality encompasses efferent and afferent modalities, facilitating communication between the gastrointestinal tract and the CNS. Specifically, sensory fibers within the vagus nerve transmit tonal information from vital organs such as the heart, lungs, stomach, and intestines to the CNS (Zhong et al., 2021). The main projection site for gut-associated vagal afferents is in the nucleus tractus solitarius (NTS); (Fülling et al., 2019). Neurons in the NTS that receive vagal sensory input can form synaptic connections with enteroendocrine cells (Kaelberer et al., 2018), and neurotransmitters and purinergic signaling transmit to the brain via specific ligand-receptor pairs to regulate microglia state and activity (Eyo and Wu, 2013). In addition, vagal efferent fibers transmit information to the intestine and release acetylcholine to reduce the inflammatory response (Pavlov and Tracey, 2005; Breit et al., 2018). A growing body of evidence indicates that microbiomes can significantly impact brain function through their interactions with the vagus nerve (Goehler et al., 2005; Bravo et al., 2011; Sgritta et al., 2019). A recent study indicated that Aβ or tau injected into the colon is transmitted to the brain via the vagus nerve, which, attenuated after vagotomy (Chen et al., 2021); stimulation of the vagus nerve can alter glutamate receptor levels involved in AD (Yesiltepe et al., 2022). Previous studies have found that stimulatory signals from the gut and vagus nerve activate the hypothalamic–pituitary–adrenal axis and promote corticosterone release (Zimomra et al., 2011), leading to changes in intestinal permeability (Moussaoui et al., 2014), dynamics (Gue et al., 1991), and mucus (Silva et al., 2014). In addition, recent studies have also revealed the importance of pathological changes in the gut-vagus-brain signaling pathway in AD (Das et al., 2022). These findings further suggest a critical role for vagal signaling as a bridge between peripheral and central crosstalk.

#### 2.2.2. Humoral signaling

Humoral signals circulate in the blood, including microbial metabolites, hormones, cytokines, and immune cells, primarily across to CNS through the intestinal and blood–brain barrier (BBB); (Paouri and Georgopoulos, 2019). Changes in the intestinal microbiome promote the production of toxic metabolites and pro-inflammatory cytokines, decreasing beneficial substances such as short-chain fatty acids (SCFAs) and other anti-inflammatory factors. This increased intestinal permeability allows the entry of pathogenic, immunostimulatory, and neuroactive substances into the body’s circulation (Xie et al., 2022) and activates local and remote immune cells, resulting in BBB dysfunction and triggering a neuroinflammatory response, especially in the hippocampal region and cerebral cortex (Kempuraj et al., 2017; Welcome, 2019). Propionate acid can contribute to immune homeostasis by regulating cellular subpopulations (Dupraz et al., 2021). Butyric acid provides sufficient energy for intestinal epithelial cells and alternative substrates for brain metabolism and upregulates the expression of tight junction proteins to enhance the integrity of the BBB (Barichello et al., 2019; Silva et al., 2020; Chen et al., 2022). In addition, SCFAs clear protein aggregates in the brain by enhancing the function of microglia (Erny et al., 2015; Wenzel et al., 2020). Several studies in AD mice have confirmed butyrate’s important role in improving microglia function and reducing Aβ deposition (Jiang et al., 2021; Liu et al., 2021). Studies have shown that SCFAs activate G-protein-coupled receptors (GPR) 43, GPR 41, and GPR 109, receptors in the intestine and brain, which can protect the BBB from oxidative stress by modulating Recombinant Cluster of Differentiation 14 signaling and activating the nuclear factor (erythroid-derived 2)-like 2 (Tan et al., 2014; Hoyles et al., 2018). Conversely, lipopolysaccharide (LPS) induces the activation of macrophages and dendritic cells to produce pro-inflammatory cytokines (Jeong et al., 2019) and is capable of interacting Toll-like receptor-4 (TLR4); Park et al., 2009). Cani et al. found that LPS can stimulate the body’s immune response by destroying intestinal epithelial cells to enter the bloodstream (Cani et al., 2008). In addition, multiple lines of evidence suggest that endotoxin promotes Aβ_42_ fiber formation (Kahn et al., 2012; Asti and Gioglio, 2014) and that microglia can be activated by endotoxin, causing central inflammation (Shen et al., 2020).

### 2.3. Gut microbiome and AD pathology

*Escherichia coli*-derived neurotoxins in *Proteobacteria* are associated with neuropathology in AD and increase the release of pro-inflammatory cytokines that induce systemic inflammation and exacerbate AD pathology (Kitazawa et al., 2005; Cattaneo et al., 2017). Many bacteria strains are capable of producing amyloid (Megur et al., 2020; Tran and Mohajeri, 2021), and the forms show similarity with the CNS amyloid (Hill and Lukiw, 2015), which can lead to an enhanced immune response and endogenous formation of neuronal amyloid in the brain (Friedland and Chapman, 2017). LPS and gram-negative *E. coli* fragments coexisted with amyloid plaques in AD brain tissue (Zhan et al., 2016). Aβ loading may initially occur in the gastrointestinal tract. Aβ_1-42_ migrates after being injected into the gastric wall, and Aβ deposition is present in the vagus nerve and brain and develops symptoms of cognitive impairment (CI; Sun Y. et al., 2020). Therefore, this could provide some basis for the movement of the GM to trigger the amyloid pathogenesis of AD. The microbiome is necessary for the normal development of hippocampal and microglial cell morphology (Luczynski et al., 2016). Recently, Liu et al. observed microbiome deficiency altered dendritic signaling integration in the Cornu Ammonis1 region of mice (Liu et al., 2022). The presence of activated microglia and reactive astrocytes in the vicinity of amyloid plaques is characteristic of AD neuroinflammation, mainly in the hippocampus (Crews and Masliah, 2010; Cattaneo et al., 2017). Moreover, in the elderly brain, microglia are dysfunctional and prone to chronic activation (Spittau, 2017). In the early pathological stages of AD, the overall microglial response can support neurons by phagocytosing Aβ fibers. However, as the disease progresses, the loss of the branching phenotype of microglia with excessive activation near plaques and aggregation of other immune cells triggers a neurotoxic environment, leading to neural network damage (Leng and Edison, 2021; Thu Thuy Nguyen and Endres, 2022). Other studies have also observed numerous roles for aberrantly regulated GM in triggering Aβ amyloidosis, neuroinflammation, and microglia regulation in AD mice (Dodiya et al., 2019).

### 2.4. Characteristic changes in AD gut microbiome

The intestinal microbiome contains two major bacterial phylotypes, the *Firmicutes,* the *Bacteroidetes*, and to a lesser extent, the *actinomycetes*, *fusobacteria*, *proteobacteria*, and *weberia* (Grenham et al., 2011). Some studies have found less diverse *Firmicutes* and *genera*, but a higher prevalence of *Proteobacteria* in AD (Nagpal et al., 2019; Sheng et al., 2021; Hung et al., 2022). Intestinal α-diversity and β-diversity are altered in patients on the AD spectrum (Hung et al., 2022). Several studies have reported a significant decrease in α-diversity in AD patients (Vogt et al., 2017; Liu P. et al., 2019), but not obvious in mild cognitive impairment (MCI) patients, and there is a similar gradual decline trend from MCI to AD (Nagpal et al., 2019). Nevertheless, results vary regarding β-diversity studies, with one Austrian study showing that malnutrition and drug intake affect the abundance of the specific microbiome, SCFAs, and butyrate production, and statins may be one of the reasons affecting beta diversity (Stadlbauer et al., 2020). A recent systematic review and meta-analysis of 11 studies included 378 normal control (NC) and 427 AD patients to analyze the impact of different countries and clinical stages on GM abundance. GM diversity was significantly lower in AD patients than in NC but not in MCI patients. Compared to NC, the AD spectrum group had an increased abundance of *Proteobacteria*, *Bifidobacterium*, and *Phascolarc* to the *Bacterium*, and a decreased abundance of *Firmicutes*, *Clostridiaceae*, *Lachnospiraceae,* and *Rikenellaceae* (Hung et al., 2022). More importantly, the abundance distribution of the *Alistipes* and *Bacteroide*s in the NC and AD differed by country (Hung et al., 2022).

Several studies have observed alterations in gut microbiology in patients with AD, but mostly with the influence of drugs and other interventions and varying disease duration. Although there is no direct evidence in clinical trials that the effects on MGBA of current medications for AD (acetylcholinesterase inhibitors or N-methyl-D-aspartate (NMDA) antagonists) have been studied (La Rosa et al., 2018), the latest study provides evidence that donepezil affects GM via amino acid pathways and sugar metabolism (Jo et al., 2022). In light of the results mentioned above, the researchers’ attention was drawn to MCI and subjective cognitive decline (SCD) patients’ gut changes. A study used 16S ribosomal ribonucleic acid (16SrRNA) sequencing to examine stool samples from 18 AD patients without treatment or intervention, 20 MCI patients, and 18 age-matched NC. The findings revealed that *Prevotella* levels were higher and *Bacteroides*, *Lachnospira*, and *Ruminiclostridium_9* levels were lower in AD patients. Additionally, greater cognitive performance was favorably correlated with *Bacteroides*, *Lachnospira*, and *Ruminiclostridium_9*, although *Prevotella* had the opposite relationship (Guo et al., 2021). Based on reduced effects such as drugs, the above results likewise support the idea of a progressive worsening of the degree of intestinal dysbiosis from MCI to the disease stage of AD (Guo et al., 2021). Based on this, Sheng et al. further explored gut microbial composition changes in SCD, the earliest symptom of preclinical AD. The study included 38 NC, 53 patients with SCD, and 14 patients with CI and compared the gut microbial composition of the three groups and the relationship with cognition using 16SrRNA technology (Sheng et al., 2021). Findings revealed a decreasing trend in the abundance of the phylum *Firmicutes*, class *Clostridia*, order *Clostridiales*, family *Ruminococcaceae*, and genus *Faecalibacterium* from NC to SCD and CI (Sheng et al., 2021). In particular, the abundance of the anti-inflammatory genus *Faecalibacterium* was significantly lower in the SCD group than in the NC group. Notably, altered bacterial taxa were associated with cognitive performance and were validated in amyloid-positive SCD participants (Sheng et al., 2021). The results further suggest that the microbiome may influence the development of amyloid pathology.

*Proteobacteria* is a major phylum of gram-negative bacteria (Zhao and Lukiw, 2018). More gram-negative bacteria have also been reported in AD patients compared to NC (Vogt et al., 2017). In addition, the abundance of *Proteobacteria* increased with increasing memory dysfunction (Hossain et al., 2019; Jeong et al., 2019). *Bacteroides* have been shown to preserve the intestinal barrier and reverse leaky gut (Hsiao et al., 2013), indicating that they may be a microbiome protective factor. *Bacteroides* levels are reduced in AD and MCI patients (Cattaneo et al., 2017; Zhuang et al., 2018). Moreover, the relative abundance of the *Actinomycete* phylum was associated with diffusion tensor imaging of the thalamus, hypothalamus, and amygdala, as well as cognitive test scores (Fernandez-Real et al., 2015). Certain *Lactobacillus* and *Bifidobacterium* secrete gamma-aminobutyric acid, which may mediate cognitive function in AD (Barrett et al., 2012), and different levels of abundance of the *Lactobacillus* family have been observed at different stages of dementia (Stadlbauer et al., 2020). Vogt et al. compared the different bacterial classifications of stool samples from AD patients versus NC and observed levels of differentially abundant genera associated with cerebrospinal fluid (CSF) biomarkers of AD pathology (Vogt et al., 2017).

In addition to the intestinal bacteria noted by most researchers, some studies have also found specific alterations in MCI patients in the genus *Fungi* and a clear correlation with AD biomarkers (Nagpal et al., 2019). More importantly, the different microbiome and fungal biota composition in 3xTg-AD mice is similar to the microbial variation found in humans, and significantly different taxa may contribute to the pathogenic cues of AD being identified (D’Argenio et al., 2022). However, there are some differences in studies regarding oral microbiology. A Canadian study used 16SrRNA sequencing to analyze stool and oral samples from AD and NC comparatively. The study found that oral microbiota exhibited greater differences between patients and controls than GM but was not associated with cognitive function (Cirstea et al., 2022). Although some results support the possibility that oral bacteria or their components may themselves invade the brain (Laugisch et al., 2018; Jungbauer et al., 2022), analyses using publicly available genome-wide association studies on periodontitis and AD failed to reveal any genetically predicted association between AD and periodontitis risk (Sun Y.-Q. et al., 2020). However, preventing and treating periodontitis and its associated inflammation could reduce neuroinflammation and prevent and treat neurodegenerative diseases (Li et al., 2022).

## 3. Microbiome-based AD biomarkers

**Figure 2:**
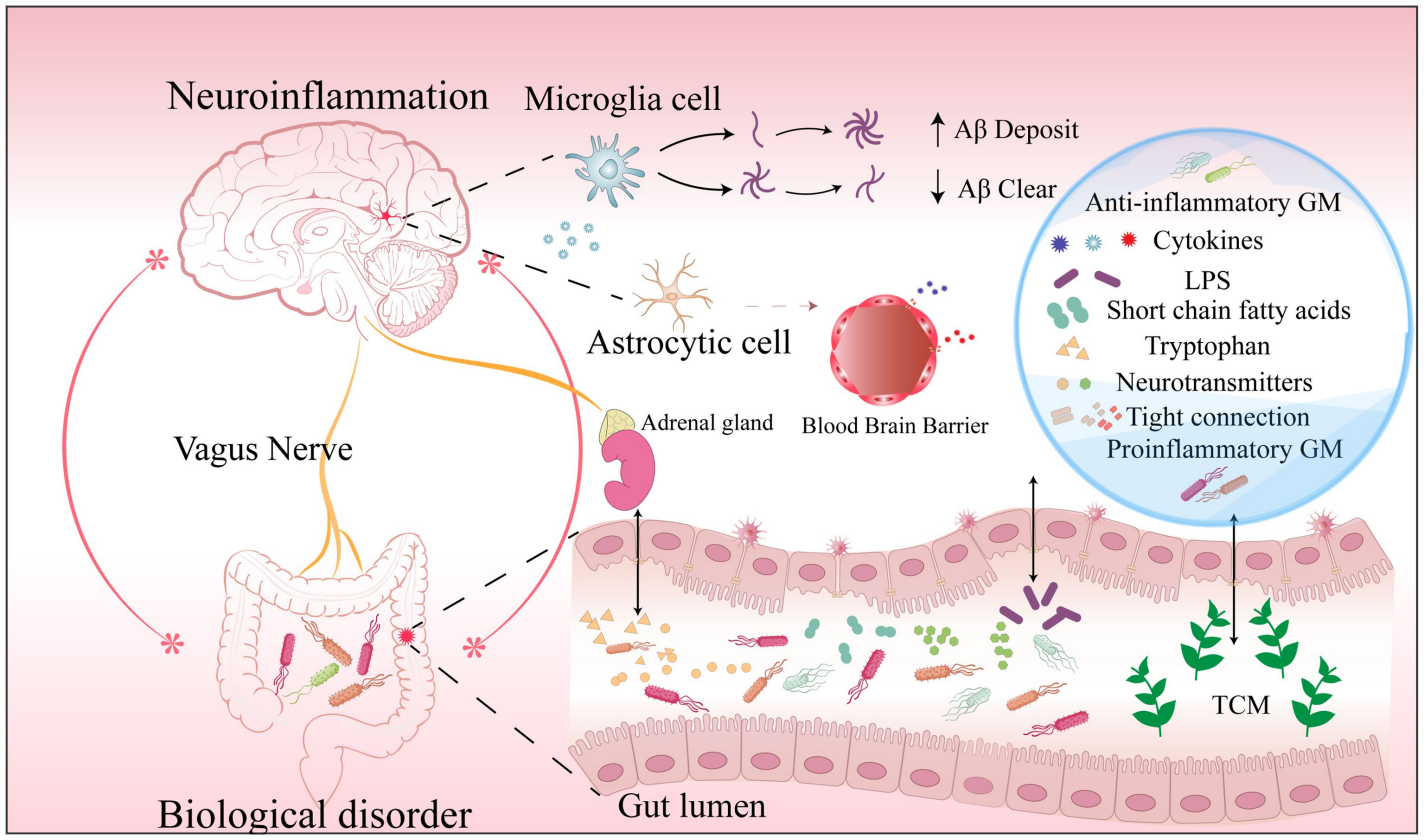
Biomarkers in the AD microbiome-gut-brain axis.Dysbiosis results in (1) a decrease in specific beneficial microbiome and metabolites and an increase in pro-inflammatory microbiome and harmful metabolites; (2) Increased intestinal permeability markers in serum and feces due to disruption of tight junctions; (3) Increased release of cytosolic inflammatory factors from microglia; (4) An increase or decrease in specific neurotransmitters involved in the development of AD. They are potential gut biomarkers in AD. Traditional Chinese Medicine ingredients can reduce AD pathology and improve cognitive function in a multi-targeted and multi-pathway manner by modulating intestinal microbes and metabolites, reshaping the intestinal barrier, and reducing neuroinflammation. Abbreviations: AD: Alzheimer’s disease; GM: gut microbiome; LPS: lipopolysaccharide; Aβ: amyloid-β protein; TCM: traditional Chinese.medicine.

The discovery of biomarkers over the past 20 years has allowed for the verification of Aβ and tau-related fluid biomarkers in clinical studies as well as their incorporation into the [AT(N)] framework for diagnosis (Mayeux, 2004; Jack et al., 2018). The [AT(N)] framework was proposed by the National Institute on Aging-Alzheimer’s Association in 2018 and focused on diagnosing AD with *in vivo* biomarkers. Biomarkers are classified as Aβ deposition, pathological tau, and neurodegeneration [AT(N)] (Jack et al., 2018). However, many pharmacological treatments targeting Aβ and tau pathological features have proven futile (Mahaman et al., 2022). This may be attributed to the difficulty of reversing the underlying neuropathological changes that have already occurred (Mahaman et al., 2022). Therefore, very early and reliable diagnostic biomarkers are urgently needed. Recently, a study found that alterations in the GM precede the development of key pathological features of AD (Chen et al., 2020; Bello-Medina et al., 2021). In addition, the apparent association between AD and specific bacterial strains has facilitated the exploration of highly promising biomarkers in GM. Gut bacterial metabolites, gut permeability, gut hormones, MGBA involvement in AD, and potential gut-based biomarkers and targeted herbal therapies are shown in Figure 2. More importantly, completely non-invasive biomarkers such as stool, saliva, or urine (Mahaman et al., 2022) may be a realistic option for large-scale population screening, early AD diagnosis, and disease progression monitoring at a low cost.

### 3.1. Biomarkers of the gut microbiome in AD

Based on many characteristic changes in the GM detected in the pre-AD period, this finding may provide clues as a diagnostic biomarker in the pre-AD period (Guo et al., 2021; Sheng et al., 2021; Verhaar et al., 2022). Hence, numerous studies have attempted to use GM for early diagnostic identification of AD.

APP/PS1 mice combining 16SrRNA gene sequencing and extensively targeted metabolomics revealed that *B. firmus*, *Rikenella*, *Clostridium* sp. *culture-27*, and deoxyuridine might be important biomarkers of AD (Feng et al., 2022). Based on the GM differences in feces and blood established in the preliminary study, Li et al. built a random forest model based on 11 genera of differences in stools and blood from AD patients and NC. Using the random forest model’s cut-off values with all different stool input genera, 28 of 30 MCI patients could be accurately identified with a sensitivity of 93% (Li et al., 2019). In addition, 33 AD, 32 amnestic MCI (aMCI), and 32 NC were examined in cross-sectional research. According to the findings, AD patients had a reduced level of fecal microbial diversity compared to aMCI patients and NC. Further, as patients advanced from NC to aMCI and AD, *Gammaproteobacteria*, *Enterobacteriales*, and *Enterobacteriaceae* gradually enriched abundance. A strong association was also discovered between the clinical severity scores of AD patients and the abundance of changed microbiomes. Notably, the model based on the abundance of *Enterobacteriaceae* bacteria could distinguish AD from aMCI and NC (Liu P. et al., 2019). This initially validates the value of intestinal biomarkers in the early identification of patients with CI and affirms the great potential of gut biomarkers. To improve the robustness of gut biomarkers, a study in 2022 simultaneously combined the latest proposed [AT(N)] framework. The study collected 34 Aβ (−) cognitively normal (CN−), 32 Aβ (+) cognitively normal (CN+), and 22 (11 MCI, 11 AD) CI patients. According to the findings, the relative abundance of bacteria that produce SCFAs reduced from CN− to CN+ and CI. Moreover, all CN subjects had a negative correlation between total brain Aβ load, plasma Aβ_42_/Aβ_40_, and 3 particular types of bacteria. It was discovered that combining plasma Aβ markers, altered GM, and cognitive ability improved discrimination between CN+ and CN− (Sheng et al., 2022). The above promising findings suggest the possibility and accuracy of GM in diagnosing AD.

Patients with newly diagnosed AD or MCI have dysbiosis of the intestinal microbiome, including a decrease in the potentially protective microbiome and an increase in the pro-inflammatory microbiome (Chen et al., 2020; Bello-Medina et al., 2021). Significant changes in gut microbial composition are observed, especially in older people, and diet may be the most important driver of change (Kumar et al., 2016). The severity of human microbiome dysbiosis shows characteristic changes in different disease stages of AD (Stadlbauer et al., 2020); an assessment of disease progression and prognosis provides some basis, suggesting that these may be meaningful biomarkers for predicting AD development (Li et al., 2019). In addition, recent studies have reported that neuroimaging of the MGBA will further improve our understanding of GM by altering neural microstructure and function in time and space (Montoro et al., 2022).

### 3.2. Biomarkers of intestinal permeability in AD

Tight junctions are an important mechanism affecting the intestinal barrier and the BBB, with the interaction of intestinal bacteria with epithelial cells being a key factor in regulating epithelial permeability by modulating tight junctions (Allam-Ndoul et al., 2020). It has been found in the last decade to be associated with AD (Schoultz and Keita, 2020). Calprotectin, a marker of intestinal inflammation (Kowalski and Mulak, 2019), is significantly increased in the CSF and brain of AD patients (Wang et al., 2014). It implies that intestinal permeability could be associated with AD pathogenesis.

Human targeting studies-the sugar absorption assay (Ménard et al., 2010), identified plasma lipopolysaccharide-binding protein (LBP) as a promising biomarker of intestinal permeability in adults (Seethaler et al., 2021). However, the markers of intestinal barrier integrity LBP and intestinal fatty acid binding protein (IFABP), are not associated with independent AD dementia, MCI, cognition, and neuropathology (Voigt et al., 2021). It is worth noting that disruption of the apical junction complex was not evaluated, and the small sample size may have influenced the results. Another study indirectly illustrated the relationship between intestinal permeability and AD. The peripheral platelet alterations are involved in the pathological process of AD and are based on the fact that C-type lectin-like receptor 2 (CLEC-2) is an activated receptor on the platelet surface (Mukaetova-Ladinska et al., 2012; Akingbade et al., 2018). Wang et al. found elevated levels of CLEC-2 and zonulin in MCI and AD patients, with a progressive increase compared to NC. Furthermore, the study identified high levels of CLEC-2 and zonulin as significant factors for decreased Mini-Mental State Examination scores (Wang X. et al., 2020). An Austrian clinical study included CI and normal population controls with serum diamine oxidase (DAO) and fecal zona pellucida protein to detect intestinal permeability and found higher levels of DAO in patients with dementia. The results suggest a correlation between dementia and increased intestinal permeability biomarkers (Stadlbauer et al., 2020). In addition, GM disorders resulted in low expression of the major tight junction proteins of the BBB (zonula occludens-1, occluding, and claudin-5) in the frontal cortex, hippocampus, and perisylvian striatum of mice (Braniste et al., 2014). More importantly, these brain regions affect memory and cognitive functions.

The findings above support a link between intestinal barrier dysfunction and AD. Some bacteria, such as *Lactobacillus plantarum*, *Escherichia coli*, or *Bifidobacterium infantis*, can improve the intestinal wall barrier by boosting the expression of tight junction-related proteins (Hakansson and Molin, 2011; Kowalski and Mulak, 2019). Increasing butyrate-producing bacteria enhanced tight junction protein expression in mice’s frontal cortex and hippocampus (Marizzoni et al., 2020). These findings give more evidence for the role of intestinal permeability in AD and may have biomarkers potential. However, it is crucial to highlight that due to the difficulties of performing sugar absorption tests in people with dementia, blood, and stool indicators are typically employed clinically instead (Stadlbauer et al., 2020). Various biomarkers associated with epithelial damage, including citrulline, IFABP, and LBP, have been used as indirect indicators of the reduced intestinal barrier (Galipeau and Verdu, 2016). However, studies have shown that even before leakage occurs, when the Notch signaling pathway involved in regulating the BBB is altered, the BBB has been disrupted, and there is already an impact on synaptic function, triggering dementia (Calderon et al., 2022). Conceivably, this will provide insights into discovering additional early markers of barrier function. Nonetheless, the mechanistic pathways that connect the gut barrier with the BBB should be studied further.

### 3.3. Biomarkers of gut metabolites AD

In AD, the intestinal microbiome and permeability characteristics have led researchers to explore the potential of gut microbial metabolites as biomarkers. The *Ruminococcaceae* metabolite isoamylamine recognizes and binds the Recombinant S100 Calcium Binding Protein A8 promoter region and promotes neuronal cell death, leading to cognitive decline in mice (Teng et al., 2022). Moreover, memory deficit in APP/PS1 mice was correlated with metabolite status. Fasudil et al. found that glutamate, hypoxanthine, thymine, hexanoyl coenzyme A, and leukotriene metabolize nucleotides, lipids, and sugars as well as inflammatory pathways involved in AD mouse-related metabolism (Yan et al., 2021). These novel results provide a valuable reference for using gut microbial metabolites as diagnostic biomarkers and therapeutic targets in clinical studies of AD. Several bacterial metabolites have been used as fecal biomarkers to characterize patients with AD. Trimethylamine N-oxide (TMAO) is a microbial-dependent metabolite (Connell et al., 2022). The study found higher TMAO levels, enhanced oxidative stress, and intestinal barrier dysfunction in MCI and AD patients (Vogt et al., 2018; Yan et al., 2021). TMAO topped the list of 56 microbial metabolites considered biomarkers of AD in a study identifying associations between AD and microbial metabolites through a web-based algorithm, successfully predicting changes in memory and fluid cognition in aging individuals (Xu and Wang, 2016). A clinical study included AD (*n* = 40), MCI (*n* = 35), and NC (*n* = 335) results show TMAO was significantly and positively correlated with p-tau, p-tau/Aβ_42_, and t-tau, neurofilament light in the CSF of AD (Vogt et al., 2018). These results suggest that TMAO may be involved in AD pathology and neurodegeneration (Vogt et al., 2018; Arrona Cardoza et al., 2022). Changes in tryptophan, methionine, tyrosine, and purine metabolism were observed in CSF from AD patients, suggesting these may be risk factors for CI (Kaddurah-Daouk et al., 2013). Some derived molecules produced by GM in the body’s circulation are also considered potential biomarkers of AD. The outer membrane of gram-negative bacteria is primarily made up of LPS (Wu L. et al., 2021), which is present in the hippocampus and superior temporal lobe neocortex of AD brains and triggers neuroinflammatory properties (Zhao et al., 2017) that have important implications for CI (Sparkman et al., 2006). In aMCI and AD patients were found to have elevated serum LPS levels compared to NC patients (Wu L. et al., 2021). In another study, the metabolomic analysis revealed significant differences between AD and NC in tryptophan metabolites, SCFAs, and staphylococcal acid, and most were associated with the altered microbiome and CI. Notably, tryptophan disorders are already present in aMCI, and SCFAs show a decreasing trend from aMCI to AD. More importantly, indole-3-pyruvic acid, a tryptophan metabolite that accurately classifies aMCI and AD with NC, is a marker for identifying and predicting AD, and five SCFAs were identified as markers for the pre-onset and progression of AD (Wu L. et al., 2021).

### 3.4. Biomarkers of gut hormones in AD

Several studies have found that hormones synthesized by intestinal cells and gut microbiomes can affect AD through neural and humoral pathways (Welcome, 2018). Notably, the microbiome may also regulate hormone levels (Yan et al., 2016; Engevik et al., 2020), suggesting that gut hormones may also be potential biomarkers of AD. Leptin affects the cortex and hippocampus and reduces Aβ in the brains of AD mice (Fewlass et al., 2004; Farr et al., 2015). In addition, chronic lateral ventricular injection of leptin effectively alleviated Aβ_1-42_-induced spatial memory deficits and reversed Aβ_1-42_-induced hippocampal late-phase LTP inhibition in rats (Tong et al., 2015). Cholecystokinin (CCK) is a satiety hormone that binds directly to CCK receptors in the hypothalamus and hindbrain to regulate appetite and is highly expressed in the hippocampus, which is essential for protecting and enhancing memory (Blevins et al., 2000; Plagman et al., 2019). A study examined the CCK levels of CSF in 287 subjects with AD and found high levels of CCK were associated with higher total CSF tau and p-tau181, associated with better cognition and more gray matter volumes in the posterior cingulate gyrus, parahippocampal gyrus, and medial prefrontal cortex. These results suggest that CCK levels may reflect compensatory protective mechanisms during AD pathology (Plagman et al., 2019). Cortisol mediates cognition through two types of receptors: Mineralocorticoid Receptors (MRs) and Glucocorticoid Receptors (GRs) (Joëls, 2006; Daskalakis et al., 2013). MRs have a higher affinity, 6–10 times higher than GRs (de Kloet et al., 1999; Joëls, 2006). In animal models, *Enterococcus faecalis* was found to promote social activity and reduce Corticosterone (CORT) levels in mice after social stress (Wu W.-L. et al., 2021); GM imbalance causes elevated CORT in AD mice (Hendrickx et al., 2021) and remodeling of the GM was able to reduce CORT levels (Liu et al., 2021). In addition, Ghrelin was found to modulate the function of hippocampal neurons and synapses in mice, thereby enhancing memory (Li et al., 2013). The above results indicate that elevated levels of stress hormones may be a potential biomarker for pre-symptomatic AD (Hendrickx et al., 2021). In humans, Sami Ouanes et al. measured cortisol and Dehydroepiandrosterone sulfate (DHEAS) levels in CSF and found that cortisol and cortisol/DHEAS ratios were positively correlated with tau and p-tau CSF levels and negatively correlated with the amygdala and insula volumes at baseline. More importantly, higher CSF cortisol and DHEAS levels predicted a more pronounced cognitive decline and disease progression over 36 months (Ouanes et al., 2022a). The higher CSF cortisol may reflect or contribute to more severe neuropsychiatric symptoms at baseline unrelated to AD pathology and more pronounced deterioration over 3 years (Ouanes et al., 2022b). These findings have important implications for identifying AD. Other studies have found reduced levels of Ghrelin messenger ribonucleic acid (mRNA) in the temporal lobe of AD patients (Gahete et al., 2010). Ghrelin may be involved in changes in neuroinflammation and cognitive function in AD (Jeon et al., 2019). These promising results further demonstrate the potential of gut hormones as biomarkers of AD.

### 3.5. Biomarkers of inflammatory factors in AD

Dysbiosis may result in the degeneration of the CNS immune response and the significant release of inflammatory cytokines that activate macrophages (Wu and Wu, 2012; Belkaid and Hand, 2014). Animal models and clinical studies have demonstrated that GM contributes to AD’s pathogenesis by regulating microglia function to involve peripheral and central inflammation processes (Bradt et al., 2000; Wu M.-L. et al., 2021). Numerous studies have demonstrated a connection between the presence and metabolism of Aβ or tau protein and the release of pro-inflammatory cytokines such as Interleukin (IL)-1, IL-6, and IL-8 by activated microglia (Teixeira et al., 2008; Breer et al., 2012; Uslu et al., 2012). IL-6 has been found to have potential properties for detecting the severity of CI in AD patients (Lai et al., 2017). Two meta-analyses found that inflammatory biomarkers such as high-sensitivity c reactive protein, transforming growth factor-beta 1 (TGF-β1), IL-1β, IL-2, IL-6, IL-12, IL-18, monocyte chemotactic protein-1 (MCP-1), MCP-3, IL-8, and interferon-γ-inducible protein 10 were consistently elevated in AD patients (Decourt et al., 2017; Su et al., 2019). The results in AD, MCI, and controls supported the view that AD and MCI are accompanied by both peripheral and CSF inflammatory responses. In addition, AD patients have higher levels of soluble tumor necrosis factor (TNF) receptor (sTNFR)-1 and sTNFR-2, which previous studies have shown to exacerbate the major pathological changes in AD (Aβ and tau pathology) (Decourt et al., 2017; Shen et al., 2019; Varesi et al., 2022a). The significant differences in peripheral inflammatory factor concentrations found in the study suggest that these markers may be useful in monitoring disease progression.

Cattaneo et al. were the first to describe patients with cerebral amyloidosis-related CI. IL-1β, C-X-C motif ligand (CXCL)-2, and NOD-like receptor family pyrin domain containing (NLRP)-3 were all positively connected with *E. coli/Shigella* abundance, *Eubacterium rectale* abundance was negatively correlated with IL-1β, CXCL-2, and NLRP3 and positively correlated with IL-10. It was also confirmed that an increased abundance of *E. coli/Shigella* and a decreased abundance of anti-inflammatory *Rectal fungi* might be associated with peripheral inflammatory status in patients with CI in brain amyloidosis (Cattaneo et al., 2017). The above study improves the robustness of microbiome-based inflammatory factors as biomarkers. Several recent studies have validated the important role of microbiome-based inflammatory biomarkers in diagnosing and detecting disease development (Shen et al., 2020; Park J.-C. et al., 2020; Park et al., 2021; Xin et al., 2021). However, larger samples and the establishment of longitudinal studies to further narrow the range of inflammatory markers remain to be addressed.

Increasing the body of evidence further validated the important role of characteristic GM, intestinal permeability, bacterial metabolites, gut hormones, and various peripheral inflammatory biomarkers in diagnosing and monitoring disease progression. But some issues need to be further addressed. First, the gut microbial species are diverse and numerous, and we have mainly focused on bacterial species; although the influence of fungi and viruses is beginning to be noted, numerous impacts and mechanisms are unclear (Ye et al., 2022). Based on the current findings, further expansion of the scope and sample size is needed to further validate the feasibility and accuracy of intestinal biomarkers in AD (Sorboni et al., 2022). Second, due to the insufficient number of countries included in the existing studies and the limitations of some animal models to which the test results can be referred (Rahman et al., 2020; Hung et al., 2022). It is not easy to obtain consistent results across various dietary, environmental, genetic, and other confounding factors (David et al., 2014; Mullane and Williams, 2020). Consider that a single biomarker may not be sufficient to describe the pathophysiology of AD fully. Finally, combining multiple markers representing different stages of disease development may be a better option (Gu et al., 2021; Zhang X. et al., 2021). Therefore, based on these issues, using intestinal biomarkers for preclinical and clinical diagnosis is still some time away. However, previous studies have focused on serum, CSF, and tissue metabolomics of AD. Because fecal biomarkers are non-invasive, readily available, and found to have great potential as biomarkers for AD, their utility can be developed in the future, perhaps as an alternative or complementary tool. Combining imaging, plasma, and other biomarkers may improve early identification rates (Mahaman et al., 2022; Sheng et al., 2022). Numerous studies have used compound biomarkers to enable more sensitive detection and early disease diagnosis. For example, diet, GM, and microRNAs can detect MCI patients (Zhang X. et al., 2021). The combination of inflammatory factors (IL-6 and interferon-γ), phosphatidylcholine, and single-chain fatty acid-producing bacteria enables early diagnosis of AD (Gu et al., 2021). Hence, gut biomarkers may be a non-invasive and cost-effective diagnostic tool for early AD screening (Table 1) summarizes the latest potential human intestinal biomarkers.

**Table 1:** Microbiome-based biomarkers in humans (↑: increase; ↓: decrease; ↑↑: significant increase; ↓↓; significant).

References	Country	Study cohort and design	Samples and methods	Results	Biomarker/s proposed
Vogt et al. (2018)	USA	AD patients (*n* = 40), MCI patients (*n* = 35), NC (*n* = 335)	Cerebrospinal TMAO levels measurement and relationships between CSF TMAO and CSF biomarkers of AD	1.**↑** TMAO in AD and MCI compared to controls 2. Elevated CSF TMAO is associated with biomarkers of AD pathology	TMAO
Liu P. et al. (2019)	China	AD patients (*n* = 33), aMCI patients (*n* = 32), NC (*n* = 32)	Phylogenetic analysis of communities using 16S rRNA MiSeq sequencing and reconstruction of the feces	1.**↓** Microbial diversity in AD compared to MCI and controls 2.**↓** *Firmicutes* in AD compared to controls 3.**↑** *Proteobacteria* in AD compared to controls 4.**↑** *Gammaproteobacteria*, *Enterobacteriales*, and *Enterobacteriaceae* in AD > MCI > controls	*Enterobacteriaceae*
Li et al. (2019)	China	AD patients (*n* = 30), MCI patients (*n* = 30), NC (*n* = 30)	Analysis of microbiome community in the feces and blood via 16SrRNA sequencing	1.**↓** Microbial diversity in AD and MCI compared to controls 2.11 genera in the feces (**↑** *Dorea*, *Lactobacillus*, *Streptococcus*, *Bifidobacterium, Blautia*, and *Escherichia*；**↓** *Alistipes*, *Bacteroides*, *Parabacteroides*, *Sutterella*, and *Paraprevotella*) and in the blood (**↑** *Propionibacterium*, *Pseudomonas*, *Glutamicibacter*, *Escherichia*, and *Acidovora*;**↓** *Acinetobacter*, *Aliihoeflea*, *Halomonas*, *Leucobacter*, *Pannonibacter*, and *Ochrobactrum*) between AD, MCI and controls	Microbial diversity
Nho et al. (2019)	USA ADNI ADMC	AD patients (*n* = 305), EMCI patients (*n* = 284), LMCI patients (*n* = 505), SMC patients (*n* = 98), NC (*n* = 370)	targeted metabolomic profiling of the Serum levels of 20 primary and secondary BA metabolite	1. Three BA (**↑** GDCA: CA, TDCA: CA, and GLCA: CDCA)signatures were associated with**↓** CSF Aβ_1-42_ (“A”) 2. Three BA (**↑** GCDCA、GLCA and TLCA) with**↑** CSF p-tau181 (“T”) 3. The CSF t-tau, glucose metabolism, and atrophy (“N”) were connected to three, twelve, and fourteen BA signatures, respectively	BA
Plagman et al. (2019)	Ames ADNI	AD patients (*n* = 66), MCI patients (*n* = 135), NC (*n* = 86)	Targeted quantitation	1.**↑** CCK was a strong relationship associated with**↑**tau levels 2.**↑** CCK was related to**↑**global and memory scores 3.**↑** CCK was related to**↑**gray matter volume	CCK
Stadlbauer et al. (2020)	Austria	Dementia patients (*n* = 23), NC (*n* = 18)	GM composition, gut barrier dysfunction, bacterial translocation, and inflammation were assessed from stool and serum samples by 16SrRNA sequencing, QIIME 2, Calypso 7.14 tools, and ELISA	1.**↓↓** *Lachnospiraceae*, genus *Lachnospiraceae NK4A136* group in dementia. 2.**↓** *Eubacterium rectale* in dementia. 3.**↑** DAO and sCD14 in dementia. 4. *Faecalibacterium prausnitzii* was associated with mild dementia; *Lactobacillus amylovorus* was associated with moderate dementia; *Clostridium clostridiforme* and *Streptococcus salivarius* were associated with Severe dementia	*Lachnospiraceae NK4A136* group, DAO, sCD14
Nagpal et al. (2020)	USA	MCI (*n* = 11), CN (*n* = 6)	16SrRNA sequencing and fungal rRNA ITS1 sequencing of fecal bacterial and mycobiome	1.**↑** *Genera Botrytis*, *Kazachstania*, *Phaeoacremonium*, *Cladosporium*, and families *Sclerotiniaceae*, *Phaffomyceteceae*, *Trichocomaceae*, *Cystofilobasidiaceae*, and *Togniniaceae* in MCI compared to CN 2. Meyerozyma in MCI< CN 3. Different correlation patterns with AD markers and GM are displayed by specific fungal species (*Debariomyce*, *Sarocladium*, *Filobasidium*, *Candida,* and *Cladosporium*)	Mycobiome signatures
Marizzoni et al. (2020)	Italy	Eighty-nine, older persons with a cognitive performance from normal to dementia	ELISA measured blood levels of LPS, and SCFAs by mass spectrometry	1.**↑** Blood LPS, acetate and valerate, pro-inflammatory cytokines, and biomarkers of endothelial dysfunction in dementia 2.**↓** Butyrate and the anti-inflammatory cytokine IL10 in dementia	SCFAs and LPS
Wu L. et al. (2021)	China	AD patients (*n* = 27), aMCI patients (*n* = 22), NC (*n* = 28)	LC/GC/MS metabolomics profiling of fecal microbiome	1.**↓** Tryptophan metabolites in MCI and, more pronounced, in AD compared to controls 2.**↓↓** SCFAs (formic acid, acetic acid, propanoic acid, 2-methylbutyric acid, and isovaleric acid) in AD compared to controls；**↓** in MCI compared to controls	Indole-3-pyruvic acid five SCFAs
Ling et al. (2021)	China	AD patients (*n* = 100), NC (*n* = 71)	16SrRNA Miseq sequencing of fecal microbiome	1.**↓** Microbial diversity in AD compared to controls 2.**↓** Butyrate-producing bacteria (*Faecalibacterium*) in AD 3.**↑** Lactate-producing bacteria (*Bifidobacterium*) in AD	*Faecalibacterium*, *Bifidobacterium*
Zhang X. et al. (2021)	China	MCI patients (*n* = 75), NC (*n* = 52)	16SrDNA gene sequencing of fecal microbiome and serum miRNA expression	1.**↓** Microbial diversity, *Faecalibacterium*, *Ruminococcaceae*, and *Alipstes* in MCI compared to controls 2.**↑** *Proteobacteria* and *Gammaproteobacteria* in MCI compared to controls	Gut microbiota composition + diet quality scores + serum miRNA
Verhaar et al. (2022)	Amsterdam	AD patients (*n* = 33), MCI patients (*n* = 21), SCD patients (*n* = 116)	16SrRNA Miseq sequencing of fecal microbiome and CSF Aβ, p-tau	1.**↑** *Clostridium leptum*,**↓** *Eubacterium* ventriosum group spp., *Lachnospiraceae* spp., *Marvinbryantia* spp., *Monoglobus* spp., *Ruminococcus* torques group spp., *Roseburia hominis*, and *Christensenellaceae R-7* spp. with higher odds of amyloid positivity 2.**↓** *Lachnospiraceae spp.*, *Lachnoclostridium spp.*, *Roseburia hominis*, and *Bilophila wadsworthia* with higher odds of positive p-tau status	SCFA-producing microorganisms
Sheng et al. (2022)	China	AD patients (*n* = 11), MCI patients (*n* = 11), CN− participants (*n* = 34), CN+ participants (*n* = 32)	16SrRNA Miseq sequencing of fecal microbiome and MSD quantifying the plasma Aβ_40_, Aβ_42_, and Aβ_42_/Aβ_40_	1.**↑↑** The relative abundance of phylum Bacteroidetes in CN+ compared to CN− 2.**↓↓** *Phylum Firmicutes* and *class Deltaproteobacteria* in CN+ compared to CN− 3. The relative abundance of *phylum Firmicutes* and its corresponding SCFA-producing bacteria in CN− > CN+ > CI	Plasma Aβ + gut microbiota
Ouanes et al. (2022a)	Switzerland	CI patients (*n* = 93), CN participants (*n* = 52)	ELISA, CLIA	1.**↑** CSF cortisol was associated with**↓**global cognitive performance and**↑**disease severity at baseline 2.**↑** Cortisol and cortisol/DHEAS ratio were associated with**↑**tau and p-tau CSF levels and**↓** amygdala and insula volumes at baseline	Cortisol and DHEAS

AD, Alzheimer disease; MCI, mild cognitive impaired; aMCI, amnestic mild cognitive impairment; SMC, significant memory concern; EMCI, early mild cognitive impairment; LMCI, late mild cognitive impairment; NC, normal controls; CN, cognitively normal; CN−, Aβ-negative cognitively normal; CN+, Aβ-positive cognitively normal; CI, cognitive impairment; GM, gut microbiome; CSF, cerebrospinal fluid; GC, gas chromatography; LC, liquid chromatography; MS, mass spectrometry; BA, Bile acid; LPS, Lipopolysaccharides; SCFAs, short chain fatty acids; TMAO, Trimethylamine N-oxide; ADNI, Alzheimer’s Disease Neuroimaging Initiative; ADMC, Alzheimer Disease Metabolomics Consortium; Aβ, amyloid-β; CA, cholic acid; CDCA, chenodeoxycholic acid; GCDCA, glycochenodeoxycholic acid; GDCA, glycodeoxycholic acid; GLCA, glycolithocholic acid; TLCA, taurolithocholic acid; CCK, Cholecystokinin; DAO, diaminooxidase; sCD14, soluble CD 14; 16SrDNA, 16Sribosomal DNA; 16SrRNA, 16Sribosomal RNA; ELISA, Enzyme linked immunosorbent assays; CLIA, chemiluminescence immunoassay; DHEAS, Dehydroepiandrosterone sulfate; MSD, meso scale discovery; USA, United States of America; p-tau, phosphorylated tau.

## 4. Computation and modeling of the gut microbiome and its implication in diagnostic biomarkers and therapeutics identification

With the rapid development of genomics, metabolomics, proteomics of blood, and other assays, the human microbiome is increasingly used as a diagnostic and therapeutic biomarkers (Wirbel et al., 2021). Given the high degree of disease heterogeneity in the population, the use of artificial intelligence (AI) and machine learning (ML) to generate predictive models allows for the more precise development of individualized treatment plans (Kaur et al., 2021). The latest large-scale meta-analysis on human gut metagenomics encompasses many diseases, different sequencing technologies, and analytical tools (Wirbel et al., 2021). The investigators developed SIAMCAT, a universal R toolbox for ML-based comparative metagenomics, and demonstrated its capability in a meta-analysis of fecal macro-genomic studies (10,803 samples). Some biomarkers were found to be shared in multiple contexts (Wirbel et al., 2021). Wang et al. constructed a context-sensitive network to prioritize and identify 8,094 potential microglia-microbial metabolite-gene-pathway-phenotype interactions in AD. The study comprehensively characterized a computational approach to complex gut-microbial metabolite-microglial-gene-pathway-phenotype-brain connections in AD by innovatively bringing together the large amount of publicly available data collected (Wang et al., 2021). By identifying gut microbial metabolites and understanding their function in AD, it may be possible to gain new knowledge about the underlying mechanisms underlying AD etiology and open new avenues for AD treatment and prevention (Wang et al., 2021). Kaur et al. proposed a framework for integrating multi-omics data by understanding the epigenetic regulation of the gut-brain axis, which could further precise detection and the development of treatments for disorders of the CNS (Kaur et al., 2021).

In proteomics, a recent study proposes a framework for microbiome computation, MetaProClust-mass spectrometry (MS) 1 (Simopoulos et al., 2022). The framework can evaluate and identify hippocampal proteomic changes in a mouse model of AD after small molecule treatment, validating that MetaProClust-MS1 can be used to screen microbiome and single species proteome and extended to any proteomics experiment (Simopoulos et al., 2022). The study suggests that MetaProClust-MS1 can be used for large-scale proteomic and clinical diagnostic screening (Simopoulos et al., 2022). In addition, numerous recent studies have begun experimenting with gut-based diagnostic models for identifying AD and obtained promising results (Jung et al., 2022; Kalecký et al., 2022; Sheng et al., 2022).

## 5. Potential targeted therapies for gut microbiome

Indeed, based on the solid evidence that the MGBA mediates AD pathology, GM is emerging as a potential target for treating AD (Seo et al., 2019). Recently, researchers have been targeting therapies to regulate GM in various ways, such as diet (Ghosh et al., 2020; Moreno-Arribas et al., 2020). In a review by Varesi et al., numerous promising dietary therapies were systematically reviewed, including the Mediterranean diet, Dietary Approaches to Stop Hypertension (DASH), the Mediterranean-DASH Neurodegenerative Delay Intervention diet, and the ketogenic diet, and intermitting fasting might be a promising protective dietary strategy for dementia (Park S. et al., 2020; Varesi et al., 2022b). In addition, fecal microbiota transplantation (FMT) is a method of repairing dysbiotic gut by re-cloning the normal microbiota into the “diseased” intestine (Gupta et al., 2016; Matheson and Holsinger, 2023). Although promising results such as restoration of microbiota composition, improved cognitive performance, and reduction in amyloid accumulation and tau expression have been observed in mice in several studies that have been reported (Dodiya et al., 2019; Sun et al., 2019), current studies in humans remain in the single digits. Therefore, more human studies are needed before pointing to FMT as a complementary therapy for AD. In the meantime, the researchers have tried other treatments, like probiotics (Athari Nik Azm et al., 2018; Bonfili et al., 2018), prebiotics (Nishikawa et al., 2021), nanotechnology (Li et al., 2021; Qu et al., 2022; Yang et al., 2022) and neurotherapy (He et al., 2021).

Currently, researchers are working to develop new drugs based on gut regulatory mechanisms. Sodium mannitrate (GV-971), a mixture of acidic linear oligosaccharides that inhibits intestinal microbiome dysbiosis and associated phenylalanine/isoleucine accumulation, treats neuroinflammation, and reverses CI, was approved for the first time in China for treatment of mild to moderate AD in 2019 and approved by the Food and Drug Administration (FDA) to carry out a phase III clinical study in 2020 (Syed, 2020). The approved anti-AD drugs by the FDA are relatively specific to a single target, like anti-amyloid drugs, acetylcholinesterase inhibitors, and NMDA antagonists. The difference is that traditional Chinese medicine (TCM) has multi-component, multi-target, and multi-pathway characteristics (Maimaiti et al., 2021). Some herbal medicines improve AD by modulating the GM and endogenous metabolites (Lu et al., 2019; Liu Y. et al., 2019), such as GuanXinNing ingredients, which exert an anti-AD effect by regulating *Akkermansia* and the *dgA-11_gut_group* and ameliorating GM dysbiosis (Zhang F. et al., 2021). Ginseng Radix Et Rhizoma and Poria were one of the main medication combinations used to treat dementia, according to earlier Chinese medical literature (Wang et al., 2018), and the combination with donepezil may increase the efficacy of improving cognitive levels (Lin and Chen, 2018). Danshen, Chuanxiong, and their active ingredients have neuroprotective effects (Zhou et al., 2016), respectively salvianolic acid A and salvianolic acid B, as well as ferulic acid, act by regulating the host metabolites (Li et al., 2020; Song et al., 2020). Moreover, G. elata contains gastrodin mechanisms of action, including modulation of neurotransmitters, exerting antioxidant and anti-inflammatory effects, inhibiting microglia activation, regulating mitochondrial cascades, and upregulating neurotrophic factors (Liu et al., 2018). Lanolin inhibits the cAMP-PKA-CREB-HDAC3 pathway in AD microglia and exerts anti-inflammatory effects (Zhang S. et al., 2022). This shows that some TCM has recently been gradually tried in treating AD, and their positive efficacy in animal models has been verified. We summarize recent microbial-based targeted therapies, focusing on traditional Chinese herbal formulas containing various natural ingredients (Table 2). However, it is undeniable that herbal attempts are still stuck in animal models, and the safety, as well as tolerability of the drugs, still need to be tested over a long period. Nonetheless, it still gives us a new direction for drug development to overcome this devastating disease.

**Table 2:** Microbiome-based targeted medication.

References	Research subjects and numbers	Type of studies	Name of the medication	Medicinal ingredients	Medication usage and durations	Outcomes
Wang et al. (2019)	Six-month-old male SAMP8; two groups of 11 mice each	RCT	LW	CA-30, an oligosaccharide, mainly composed of stachyose and mannotriose	Intragastric administration of CA-30 (0.1 mL/10 g body weight) once daily for 199 days	Ameliorated the intestinal microbiome, rebalanced the NIM network, and improved cognitive impairments
Wang T. et al. (2020)	The placebo (*n* = 85) 600-mg (*n* = 84) 900-mg groups (*n* = 86)	Multicenter, randomized, double-blind, placebo parallel controlled phase II clinical trial	GV-971	Sodium oligomannate, a marine-derived oligosaccharide	Three capsule 150-mg GV-971 capsules b.i.d. (900-mg group), two 150-mg GV-971 capsules plus one placebo capsule b.i.d. (600-mg group), or three placebo capsules b.i.d. for 24 weeks	Safe and well tolerated, carry out a phase III clinical trial for GV-971 with the chosen dosage of 900 mg
Zhang F. et al. (2021)	WHBE rabbits aged 2–3 months (2–2.5 kg) NC group (normal chow, *n* = 6), the AD group (2% cholesterol diet, *n* = 6), and the GXN group (2% cholesterol diet + GXN intervention, *n* = 6)	RCT	GXN	composed of two Chinese herbs: Salvia miltiorrhiza Bge and Ligusticum chuanxiong Hort	Orally administered 250 mg/kg GXN daily for 12 weeks	Improving GM, host metabolites, and neuronal apoptosis, reducing cholesterol levels and Aβ deposition, and improving memory and behaviors
Xiao et al. (2021)	Mild-to-moderate AD the placebo (*n* = 410) GV-971–900 mg (*n* = 408)	A phase 3, double-blind, placebo-controlled trial	GV-971	Sodium oligomannate, a marine-derived oligosaccharide	450 mg of GV-971 or a placebo twice daily for 36 weeks	Safe, well-tolerated, and significantly effective and persistent in enhancing cognition in observation periods
Gu et al. (2021)	8-month-old APP/PS1 transgenic mice (*n* = 11) WT mice (*n* = 11)	RCT	HLJDD	TCM is made up of the following components in the following proportions: Fructus Gardeniae (Fg), Cortex phellodendri (Cp), Radix scutellariae (Rs), and Rhizoma coptidis (Rc) in a weight ratio of 3:2:2:3	HLJDD was continuously administered for 4 months, along with H-L (172 mg/kg/day) and H-H (344 mg/kg/day)	Improves intestinal dysregulation and reduces Aβ aggregation, which lowers neuroinflammation and improves cognition
Xiong et al. (2022)	Normal group (*n* = 14) model group (*n* = 14) sham operation group (*n* = 14) low-dose group (*n* = 14) medium-dose group (*n* = 14) high-dose group (*n* = 14) positive groups (*n* = 14)	RCT	QWF (a classic Chinese formulation)	Contains seven herbal medicines, including the bark of Cinnamomum cassia Presl, the root of Polygala tenuifolia Willd., and the sclerotium of Poria cocos (Schw.) Wolf, and the root and rhizome of Panax ginseng C. A. Mey, the root and rhizome of Acorus tatarinowii Schott, the root of Asparagus cochinchinensis (Lour.) Merr, and the root bark of Lycium chinense Mill	Daily with QWF/ 4 weeks., 5.6 g/kg/day (low dose), 11.2 g/kg/day (medium dose), 22.4 g/kg/day (high dose)	Decrease the deposition of Aβ_1-42_, downregulate the expression of NF-κB, TNF-α, and IL-6, suppress pro-inflammatory factors, and modulate the intestinal microbiome
Zhang S. et al. (2022)	WT group (*n* = 7) Tg group (*n* = 10) LS group (*n* = 9) HS (100 mg/kg) group (*n* = 9)	RCT	Erigeron breviscapus (Chinese herb)	Scutellarin, a flavonoid purified	Oral gavage (0.3 mL/day) every afternoon on weekdays/2 months (20 mg/kg)	Improved pathology, neuroinflammation, and cognitive deficits and reversed the association between acetylated histone 3 and IL-1β promoter
Fasina et al. (2022)	NC (*n* = 10) Dgal group (*n* = 10) Dgal + Done 3 mg/kg group (*n* = 10) Dgal + Gas 3 mg/kg (*n* = 10) Dgal + Gas 90 mg/kg (*n* = 10) Dgal + Gas 210 mg/kg groups (*n* = 10)	RCT	Gastrodia elata	Gas (principal), parishin, p-hydroxybenzyl alcohol, vanillin, and vanillyl alcohol compounds	Water was administered to the control and Dgal groups, while the other groups received the corresponding medication dosage orally once daily for 9 weeks	Targeting the MGBA and mitigating neuron inflammation. Improves the memory

CRS, cross-sectional study; RCT, randomized controlled trial; LW, Liuwei Dihuang decoction; SAMP8, senescence-accelerated mouse prone 8; NC, normal control; NIM, neuroendocrine immunomodulation; GXN, GuanXinNing Tablet; WHBE, white hair and black eyes; HLJDD, Huanglian Jiedu decoction; TCM, Traditional Chinese medicine; WT, wild-type mice; H-H, HLJDD with high dosage; H-L, HLJDD with low dosage; QWF, Qisheng Wan formula; IL-1β, interleukin-1β; Tg, The APP/PS1 transgenic mice; LS, low concentration; HS, high concentration; Gas, gastrodin; Dgal, D-galactose; Done, donepezil; GM, gut microbiome; MGBA, microbiome-gut-brain axis; NF-κB, nuclear factor-κB.

## 6. Conclusion

GM exerts a significant role in AD pathogenesis by affecting brain function directly or indirectly. In this review, we address dysbiosis and identify the important roles of GM, permeability alterations, bacterial metabolites, gut hormones, and inflammatory factors in AD as potential preclinical and clinical biomarkers and potential target treatment effects. A single biomarker may not be enough to comprehend AD’s pathophysiology. The early and precise identification of preclinical AD and monitoring of disease progression at different stages, using the combination of different sensitive intestinal biomarkers and composite biomarkers of plasma, neuroimages, saliva, and other binding substances, may be one of the tools for a more precise diagnosis. However, the reproducibility and accuracy of gut biomarker applications remain to be addressed and need to be further validated on a larger scale and with larger sample sizes. Nevertheless, the potentially reliable intestinal biomarkers in human AD clinical studies will provide insight into the future of early, accurate, non-invasive diagnosis. Notably, applying AI and ML makes applying gut biomarkers a vast prospect. Identifying possible targeted therapeutic efficacy in herbs that modify the balance of the intestinal environment to alleviate AD pathology and enhance cognitive-behavioral symptoms provides us with optimism in the battle against AD. Future attempts at combining gut modulation with other drugs for AD pathogenesis may yield new and surprising efficacy and provide new ideas for future AD treatment.

## Author contributions

YZ and MA-N conception and literature review. CoD writing–original draft and illustrated paintings. LZ and ChD supervised and edited the manuscript. All authors contributed to the article and approved the submitted version.

## Conflict of interest

The authors declare that the research was conducted in the absence of any commercial or financial relationships that could be construed as a potential conflict of interest.

## Publisher’s note

All claims expressed in this article are solely those of the authors and do not necessarily represent those of their affiliated organizations, or those of the publisher, the editors and the reviewers. Any product that may be evaluated in this article, or claim that may be made by its manufacturer, is not guaranteed or endorsed by the publisher.
