# Supplementary material for: The potential of the gut microbiome for identifying Alzheimer’s disease diagnostic biomarkers and future therapies
Source: Front Neurosci. 2023 Apr 27;17:1130730. doi: 10.3389/fnins.2023.1130730 (PMC10174259; doi:10.3389/fnins.2023.1130730)
Supplement: Supplementary file 1 [file Table_1.DOCX]

**Table 1.** Microbiome-based biomarkers in humans

| **References** | **Country** | **Study Cohort and Design** | **Samples and methods** | **Results** | **Biomarker/s**  **Proposed** |
| --- | --- | --- | --- | --- | --- |
| **Vogt et al., 2018**(Vogt et al., 2018) | USA | AD patients (n = 40), MCI patients (n = 35), NC (n = 335) | Cerebrospinal TMAO levels measurement and relationships between CSF TMAO and CSF biomarkers of AD | 1.**↑** TMAO in AD and MCI compared to controls  2. Elevated CSF TMAO is associated with biomarkers of AD pathology | TMAO |
| **Liu et al., 2019**(Liu et al., 2019a) | China | AD patients (n = 33), aMCI patients (n = 32), NC (n = 32) | Phylogenetic analysis of communities using 16S rRNA MiSeq sequencing and reconstruction of the feces | 1.**↓** Microbial diversity in AD compared to MCI and controls  2.**↓** *Firmicutes* in AD compared to controls  3.**↑** *Proteobacteria* in AD compared to controls  4.**↑** *Gammaproteobacteria*, *Enterobacteriales*, and *Enterobacteriaceae* in AD > MCI > controls | *Enterobacteriaceae* |
| **Li et al., 2019**(Li et al., 2019) | China | AD patients (n = 30), MCI patients (n = 30),  NC (n = 30) | Analysis of microbiome community in the feces and blood via 16S rRNA sequencing | 1.**↓** Microbial diversity in AD and MCI compared to controls  2.11 genera in the feces（**↑** *Dorea*, *Lactobacillus*, *Streptococcus*, *Bifidobacterium, Blautia*, and *Escherichia*；**↓** *Alistipes*, *Bacteroides*, *Parabacteroides*, *Sutterella*, and *Paraprevotella*） and in the blood（**↑** *Propionibacterium*, *Pseudomonas*, *Glutamicibacter*, *Escherichia*, and *Acidovora*; **↓** *Acinetobacter*, *Aliihoeflea*, *Halomonas*, *Leucobacter*, *Pannonibacter*, and *Ochrobactrum*） between AD, MCI and controls. | Microbial diversity |
| **References** | **Country** | **Study Cohort and Design** | **Samples and methods** | **Results** | **Biomarker/s**  **Proposed** |
| **Nho et al.,**  **2019**(Nho et al., 2019) | USA  ADNI  ADMC | AD patients (n = 305), EMCI patients (n = 284), LMCI patients (n = 505), SMC patients (n = 98), NC  (n =370) | targeted metabolomic profiling of the Serum levels of 20 primary and secondary BA metabolite | 1. Three BA (**↑** GDCA: CA, TDCA :CA, and GLCA :CDCA)signatures were associated with **↓** CSF Aβ_1-42_ (“A”)  2. Three BA(**↑** GCDCA、GLCA and TLCA) with **↑** CSF p-tau181 (“T”)  3. The CSF t-tau, glucose metabolism, and atrophy ("N") were connected to three, twelve, and fourteen BA signatures, respectively. | BA |
| **Plagman** **et al.,**  **2019**(Plagman et al., 2019) | Ames  ADNI | AD patients (n = 66), MCI patients (n = 135),  NC (n = 86) | targeted quantitation | 1.**↑** CCK was a strong relationship associated with **↑**tau levels  2.**↑** CCK was related to **↑**global and memory scores  3.**↑** CCK was related to**↑**gray matter volume. | CCK |
| **Stadlbauer et al.,2020**(Stadlbauer et al., 2020) | Austria | Dementia patients(n=23),  NC (n =18) | GM composition, gut barrier dysfunction, bacterial translocation, and inflammation were assessed from stool and serum samples by 16S rRNA sequencing, QIIME 2, Calypso 7.14 tools, and ELISA | 1.**↓↓** *Lachnospiraceae*, genus *Lachnospiraceae NK4A136* group in dementia.  2.**↓** *Eubacterium* *rectale* in dementia.  3.**↑** DAO and sCD14 in dementia.  4. *Faecalibacterium prausnitzii* was associated with mild dementia; *Lactobacillus amylovorus* was associated with moderate dementia; *Clostridium clostridiforme* and *Streptococcus salivarius* were associated with Severe dementia | *Lachnospiraceae NK4A136* group, DAO, sCD14 |
| **Nagpal et al.,2020**(Nagpal et al., 2020) | USA | MCI (n =11),  CN (n =6) | 16S rRNA sequencing and fungal rRNA ITS1 sequencing of fecal bacterial and mycobiome | 1.**↑** *Genera* *Botrytis*, *Kazachstania*, *Phaeoacremonium*, *Cladosporium*, and families *Sclerotiniaceae*, *Phaffomyceteceae*, *Trichocomaceae*, *Cystofilobasidiaceae*, and *Togniniaceae* in MCI compared to CN  2. Meyerozyma in MCI＜ CN  3. Different correlation patterns with AD markers and GM are displayed by specific fungal species (*Debariomyce*, *Sarocladium*, *Filobasidium*, *Candida,* and *Cladosporium*) | Mycobiome signatures |
| **References** | **Country** | **Study Cohort and Design** | **Samples and methods** | **Results** | **Biomarker/s**  **Proposed** |
| **Marizzoni et al.,**  2020(Marizzoni et al., 2020) | Italy | Eighty-nine, older persons with a cognitive performance from normal to dementia | ELISA measured blood levels of LPS, and SCFAs by mass spectrometry | 1.**↑** Blood LPS, acetate and valerate, pro-inflammatory cytokines, and biomarkers of endothelial dysfunction in dementia  2.**↓** Butyrate and the anti-inflammatory cytokine IL10 in dementia | SCFAs and LPS |
| **Wu et al.,**  **2021**(Wu et al., 2021a) | China | AD patients (n = 27),  aMCI patients (n = 22),  NC (n = 28) | LC/GC/MS metabolomics profiling of fecal microbiome | 1.**↓** Tryptophan metabolites in MCI and, more pronounced, in AD compared to controls  2.**↓↓**  SCFAs (formic acid, acetic acid, propanoic acid, 2-methylbutyric acid, and isovaleric acid) in AD compared to controls；**↓** in MCI compared to controls | Indole-3-pyruvic acid five SCFAs |
| **Ling et al.,**  **2021**(Ling et al., 2021) | China | AD patients (n = 100),  NC (n = 71) | 16S rRNA Miseq sequencing of fecal microbiome | 1.**↓** Microbial diversity in AD compared to controls  2.**↓** Butyrate-producing bacteria (*Faecalibacterium*) in AD  3.**↑** Lactate-producing bacteria (*Bifidobacterium*) in AD | *Faecalibacterium*, *Bifidobacterium* |
| **Zhang et al.,2021**(Zhang et al., 2021b) | China | MCI patients (n = 75),  NC (n = 52) | 16SrDNA gene sequencing of fecal microbiome and serum miRNA expression | 1.**↓** Microbial diversity, *Faecalibacterium*, *Ruminococcaceae*, and *Alipstes* in MCI compared to controls  2.**↑** *Proteobacteria* and *Gammaproteobacteria* in MCI compared to controls | Gut microbiota composition+diet quality scores + serum miRNA |
| **Verhaar et al.,2022**(Verhaar et al., 2022) | Amsterdam | AD patients (n = 33),  MCI patients (n = 21),  SCD patients (n = 116) | 16SrRNA Miseq sequencing of fecal microbiome and CSF Aβ, p-tau | 1.**↑** *Clostridium leptum*, **↓** *Eubacterium* ventriosum group spp., *Lachnospiraceae* spp., *Marvinbryantia* spp., *Monoglobus* spp., *Ruminococcus* torques group spp., *Roseburia hominis*, and *Christensenellaceae R-7* spp. with higher odds of amyloid positivity  2.**↓** *Lachnospiraceae spp.*, *Lachnoclostridium spp.*, *Roseburia hominis*, and *Bilophila wadsworthia* with higher odds of positive p-tau status. | SCFA-producing microorganisms |
| **Sheng et al.,2022**(Sheng et al., 2022) | China | AD patients (n = 11),  MCI patients (n = 11),  CN− participants (n = 34),  CN+ participants (n = 32) | 16SrRNA Miseq sequencing of fecal microbiome and MSD quantifying the plasma Aβ_40_, Aβ_42_, and Aβ_42_/Aβ_40_ | 1.**↑↑** The relative abundance of phylum Bacteroidetes in CN+ compared to CN−  2.**↓↓** *Phylum Firmicutes* and *class Deltaproteobacteria* in CN+ compared to CN−  3. The relative abundance of *phylum Firmicutes* and its corresponding SCFA-producing bacteria in CN− > CN+ >CI. | Plasma Aβ+gut microbiota |
| **Ouanes et al.,2022**(Ouanes et al., 2022a) | Switzerland | CI patients (n = 93),  CN participants (n = 52) | ELISA, CLIA | 1.**↑** CSF cortisol was associated with **↓**global cognitive performance and**↑**disease severity at baseline.  2.**↑** Cortisol and cortisol/DHEAS ratio were associated with **↑**tau and p-tau CSF levels and **↓** amygdala and insula volumes at baseline. | Cortisol and DHEAS |

**Table 1.** Microbiome-based biomarkers in humans （↑: increase; ↓: decrease; ↑↑: significant increase; ↓↓; significant）. Abbreviations: AD: Alzheimer disease; MCI: mild cognitive impaired; aMCI: amnestic mild cognitive impairment; SMC: significant memory concern; EMCI, early mild cognitive impairment; LMCI, late mild cognitive impairment; NC: normal controls; CN: cognitively normal; CN−: Aβ-negative cognitively normal ; CN+ : Aβ-positive cognitively normal; CI: cognitive impairment; GM: gut microbiome; CSF: cerebrospinal fluid; GC: gas chromatography; LC = liquid chromatography; MS: mass spectrometry; BA: Bile acid; LPS: Lipopolysaccharides; SCFAs: short chain fatty acids; TMAO: Trimethylamine N-oxide; ADNI: Alzheimer’s Disease Neuroimaging Initiative; ADMC: Alzheimer Disease Metabolomics Consortium; Aβ: amyloid-β; CA: cholic acid; CDCA: chenodeoxycholic acid; GCDCA: glycochenodeoxycholic acid; GDCA: glycodeoxycholic acid; GLCA: glycolithocholic acid; TLCA: taurolithocholic acid; CCK: Cholecystokinin; DAO: diaminooxidase; sCD14: soluble CD 14; 16SrDNA: 16Sribosomal DNA; 16SrRNA:16Sribosomal RNA; ELISA: Enzyme linked immunosorbent assays; CLIA: chemiluminescence immunoassay; DHEAS: Dehydroepiandrosterone sulfate; MSD: meso scale discovery; USA: United States of America; p-tau: phosphorylated tau.
