# Supplementary material for: The potential of the gut microbiome for identifying Alzheimer’s disease diagnostic biomarkers and future therapies
Source: Front Neurosci. 2023 Apr 27;17:1130730. doi: 10.3389/fnins.2023.1130730 (PMC10174259; doi:10.3389/fnins.2023.1130730)
Supplement: Supplementary file 2 [file Table_2.DOCX]

**Table 2.** Microbiome-based targeted treatments.

| **References** | **Research Subjects and Numbers** | **Type of Studies** | **Name of the Medication** | **Medicinal ingredients** | **Medication usage and durations** | **Outcomes** |
| --- | --- | --- | --- | --- | --- | --- |
| Wang et al.,  2019(Wang et al., 2019) | Six-month-old male SAMP8;  two groups of 11 mice each | RCT | LW | CA-30, an oligosaccharide, mainly composed of stachyose and mannotriose | Intragastric administration of CA-30 (0.1 mL/10 g body weight) once daily for 199 days. | Ameliorated the intestinal microbiome, rebalanced the NIM network, and improved cognitive impairments. |
| Wang et al.,  2020(Wang et al., 2020a) | the placebo(n=85)  600-mg(n=84)  900-mg groups(n=86) | multicenter, randomized, double-blind, placebo parallel controlled phase II clinical trial | GV-971 | Sodium oligomannate, a marine-derived oligosaccharide, | Three capsule 150-mg GV-971 capsules b.i.d. (900-mg group), two 150-mg GV-971 capsules plus one placebo capsule b.i.d. (600-mg group), or three placebo capsules b.i.d. for 24 weeks | Safe and well tolerated, carry out a phase III clinical trial for GV-971 with the chosen dosage of 900 mg. |
| Zhang et al.,  2021(Zhang et al., 2021a) | WHBE rabbits aged 2 to 3 months (2–2.5 kg)  NC group (normal chow, n = 6), the AD group (2% cholesterol diet, n =6), and the GXN group (2% cholesterol diet + GXN intervention, n = 6) | RCT | GXN | composed of two Chinese herbs: Salvia miltiorrhiza Bge and Ligusticum chuanxiong Hort | Orally administered 250 mg/kg GXN daily for 12 weeks. | Improving GM, host metabolites, and neuronal apoptosis, reducing cholesterol levels and Aβ deposition, and improving memory and behaviors. |
| Xiao et al.,  2021(Xiao et al., 2021) | mild-to-moderate AD  the placebo (n=410)  GV-971-900mg (n=408) | a phase 3, double-blind, placebo-controlled trial | GV-971 | Sodium oligomannate, a marine-derived oligosaccharide | 450 mg of GV-971 or a placebo twice daily for 36 weeks | Safe, well-tolerated, and significantly effective and persistent in enhancing cognition in observation periods. |
| Gu et al.,  2021(Gu et al., 2021) | 8-month-old APP/PS1 transgenic mice (n=11)  WT mice (n=11) | RCT | HLJDD | TCM is made up of the following components in the following proportions: Fructus Gardeniae (Fg), Cortex phellodendri (Cp), Radix scutellariae (Rs), and Rhizoma coptidis (Rc) in a weight ratio of 3:2:2:3. | HLJDD was continuously administered for 4 months, along with H-L (172 mg/kg/day) and H-H (344 mg/kg/day). | Improves intestinal dysregulation and reduces Aβ aggregation, which lowers neuroinflammation and improves cognition. |
| Xiong et al.,  2022(Xiong et al., 2022) | Normal group (n=14)  model group (n=14)  sham operation group (n=14)  low-dose group (n=14)  medium-dose group (n=14)  high-dose group (n=14)  positive groups (n=14) | RCT | QWF (a classic Chinese formulation) | contains seven herbal medicines, including the bark of Cinnamomum cassia Presl, the root of Polygala tenuifolia Willd., and the sclerotium of Poria cocos (Schw.) Wolf, and the root and rhizome of Panax ginseng C. A. Mey, the root and rhizome of Acorus tatarinowii Schott, the root of Asparagus cochinchinensis (Lour.) Merr, and the root bark of Lycium chinense Mill. | Daily with QWF/ 4 weeks., 5.6 g/kg/d (low dose), 11.2 g/kg/d (medium dose), 22.4 g/kg/d (high dose). | Decrease the deposition of Aβ_1-42_, downregulate the expression of NF- κB, TNF- α, and IL-6, suppress pro-inflammatory factors, and modulate the intestinal microbiome. |
| Zhang et al.,  2022(Zhang et al., 2022b) | WT group (n=7)  Tg group (n=10)  LS group (n=9)  HS (100 mg/kg) group (n=9) | RCT | Erigeron breviscapus (Chinese herb) | Scutellarin, a flavonoid purified | Oral gavage (0.3 ml/day)  every afternoon on weekdays/ 2 months  (20 mg/kg) | Improved pathology, neuroinflammation, and cognitive deficits and reversed the association between acetylated histone 3 and IL-1β promoter. |
| Fasina et al.,  2022(Fasina et al., 2022) | NC (n=10)  Dgal group (n=10)  Dgal + Done 3 mg/kg group (n=10)  Dgal + Gas 3 mg/kg (n=10)  Dgal + Gas 90 mg/kg (n=10)  Dgal + Gas 210 mg/kg groups (n=10) | RCT | Gastrodia elata | Gas(principal), parishin, p-hydroxybenzyl alcohol, vanillin, and vanillyl alcohol compounds | Water was administered to the control and Dgal groups, while the other groups received the corresponding medication dosage orally once daily for nine weeks. | Targeting the MGBA and mitigating neuron inflammation. Improves the memory. |

**Table 2.** Microbiome-based targeted medication (abbreviations: CRS: cross-sectional study; RCT: randomized controlled trial; LW: Liuwei Dihuang decoction; SAMP8: senescence-accelerated mouse prone 8; NC: normal control; NIM: neuroendocrine immunomodulation; GXN: GuanXinNing Tablet; WHBE: white hair and black eyes; HLJDD: Huanglian Jiedu decoction; TCM: Traditional Chinese medicine; WT: wild-type mice; H-H: HLJDD with high dosage; H-L: HLJDD with low dosage; QWF: Qisheng Wan formula; IL-1β: interleukin-1β; Tg: The APP/PS1 transgenic mice; LS: low concentration; HS: high concentration; Gas: gastrodin; Dgal: D-galactose; Done: donepezil; GM: gut microbiome; MGBA: microbiome-gut-brain axis; NF-κB: nuclear factor-κB ).
